# Mas Receptor Blockade Promotes Renal Vascular Response to Ang II after Partial Kidney Ischemia/Reperfusion in a Two-Kidney-One-Clip Hypertensive Rats Model

**DOI:** 10.1155/2021/6618061

**Published:** 2021-04-20

**Authors:** Farzaneh Karimi, Mehdi Nematbakhsh

**Affiliations:** ^1^Water and Electrolytes Research Center, Isfahan University of Medical Sciences, Isfahan, Iran; ^2^Department of Physiology, Isfahan University of Medical Sciences, Isfahan, Iran; ^3^Isfahan MN Institute of Basic and Applied Sciences Research, Isfahan, Iran

## Abstract

**Background:**

Partial kidney ischemia-reperfusion (IR) injury is the principal cause of acute kidney injury. The renin-angiotensin system (RAS) and hypertension also may be influenced by renal IR injury. In two models of partial renal IR with and without ischemia preconditioning (IPC) and using Mas receptor (MasR) blockade, A779 or its vehicle, the renal vascular responses to angiotensin II (Ang II) administration in two-kidney-one-clip (2K1C) hypertensive rats were determined.

**Methods:**

Thirty-seven 2K1C male Wistar rats with systolic blood pressure ≥150 mmHg were randomly divided into three groups; sham, IR, and IPC + IR. The animals in the sham group underwent surgical procedures except partial IR. The rats in the IR group underwent 45 min partial kidney ischemia, and the animals in the IPC + IR group underwent two 5 min cycles of partial kidney ischemia followed by 10 min reperfusion and partial kidney ischemia for 45 min. The renal vascular responses to graded Ang II (30, 100, 300, and 1000 ng kg^−1^.min^−1^) infusion using A779 or its vehicle were measured at constant renal perfusion pressure.

**Results:**

Four weeks after 2K1C implementation, the intravenous infusion of graded Ang II resulted in dose-related increases in mean arterial pressure (MAP) (*P*_dose_ < 0.0001) that was not different significantly between the groups. No significant differences were detected between the groups in renal blood flow (RBF) or renal vascular resistance (RVR) responses to Ang II infusion when MasR was not blocked. However, by MasR blockade, these responses were increased in IR and IPC + IR groups that were significantly different from the sham group (*P* < 0.05). For example, infusion of Ang II at dose 1000 ng kg^−1^.min^−1^ resulted in decreased RBF percentage change (RBF%) from the baseline to 17.5 ± 1.9%, 39.7 ± 3.8%, and 31.0 ± 3.4% in sham, IR, and IPC + IR, respectively.

**Conclusion:**

These data revealed the important role of MasR after partial kidney IR in the responses of RBF and RVR to Ang II administration in 2K1C hypertensive rats.

## 1. Introduction

Kidney partial ischemia-reperfusion (IR) injury is defined as a reduction of the renal blood flow (RBF) followed by the recovery of RBF and reoxygenation [1]. IR promotes a cascade of molecular events that lead to renal vascular and tubular damage and, ultimately, acute kidney injury (AKI) [2–4]. Ischemia preconditioning (IPC) against IR also was recognized as a process to minimize the kidney damage caused by IR [5]. IPC is described as short, transient, and nonlethal ischemia periods and subsequent reperfusion which is performed before IR injury and can protect the kidney against long-term ischemia [6–8].

The renin-angiotensin system (RAS) plays an important role in the regulation of blood pressure and fluid and electrolyte homeostasis [9, 10]. Hypertension and RAS are influenced by IR [11–13]. The RAS consists of two counterregulatory axes, divided into the conventional axis and includes angiotensin II (Ang II), angiotensin-converting enzyme (ACE), Ang II type 1 receptor (AT_1_R), and the nonconventional axis composed of Ang1-7 ACE2, Ang II type 2 receptor (AT_2_R), and Mas receptor (MasR) [13]. The biological effects of Ang1–7 are mainly mediated by specific receptor of MasR, and it is known clearly that Ang 1–7 and Ang II perform different actions in the renal vasculature [14]. MasR as an antagonist of AT_1_R has renoprotective effect in a number of kidney disorders, including AKI, hypertensive and diabetic nephropathy, glomerulonephritis, and tubulointerstitial fibrosis [14, 15]. In contrast to the negative effects of Ang II on the renal circulation, Ang1–7 as a renoprotective agent improves endothelial function, which increases RBF and decreases renal vascular resistance (RVR) [16].

Two-kidney-one-clip (2K1C) as a model of renin-dependent hypertension and IR alter the balance between two axes of RAS [17–19]. AT_2_R and MasR expression decrease in 2K1C [19, 20], while IR increased the intrarenal levels of Ang II, AT_2_R, and MasR [12, 21, 22] and decreased renal cortical Ang II binding [23]. Therefore, MasR activity during IR may be a compensatory mechanism to protect the kidney against IR [12].

There also is an interaction between the components of RAS. So, MasR not only acts as a specific receptor for Ang 1–7 but also interacts with AT_1_R and AT_2_R and alters the renal vascular responses to Ang II [24, 25]. In addition, IR and hypertension are risk factors for AKI, the delayed function of transplanted kidney, transplant rejection, and patient survival [26, 27].

Accordingly, due to the alteration of RAS components by IR and hypertension and interaction between MasR with other receptors of RAS, we hypothesized that MasR may alter renal vascular response to Ang II administration in hypertensive rats that underwent IR with and without IPC. To prove the hypothesis, rats were subjected to 2K1C, 4 weeks later, under anesthesia, IR with and without IPC was implemented, and renal vascular responses to Ang II administration in the presence or absence of MasR were determined.

## 2. Materials and Methods

### 2.1. Animals

Male Wistar rats (*n* = 37, 246 ± 5 g, 7–8 weeks) were used in this study. The animals were obtained from the Water and Electrolyte Research Center, Isfahan University of Medical Sciences, Isfahan, Iran. The animals were kept in polyacrylic cages and maintained under standard laboratory conditions (temperature, 23 ± 2°C) with a 12 : 12 h light/dark cycle and free access to food and water. The protocol of this research was approved in advance by the Ethics Committee of Isfahan University of Medical Sciences (code# IR.MUI.REC.1397.345).

### 2.2. Surgical Preparation to Induce the 2K1C Model

Rats were anesthetized with chloral hydrate (450 mg/kg, i.p.) and xylazine (10 mg/kg, i.p.). A 5 cm longitudinal incision was made on the right flank. The right kidney was exposed, and the renal artery was isolated from the renal vein. To induce 2K1C renovascular hypertension, the renal artery was clipped by placing a U-shaped silver clip (0.2 mm inner diameter) around it [28] to partially reduce RBF [29, 30]. The incision was sutured, and the animal was allowed to recover for four weeks.

### 2.3. Experimental Surgery

Four weeks after renal artery clipping, the animals were anesthetized with urethane (1.7 g·kg^−1^ i.p; Merck, Germany). After intubation of the trachea, the left jugular vein was catheterized by using a polyethylene catheter (PE 9658, Microtube Extrusions, North Rocks NSW, Australia). Also, polyethylene catheters were inserted into the carotid and femoral arteries. Then, the animals were located in a lateral position, and after a transverse incision on the left side, the left kidney was isolated from the surrounding tissues and was placed in the kidney cup. The renal artery was isolated, and an ultrasonic probe was placed around it (TRANSONIC MAO.7 PSB, Flowprobe, USA). Also, the abdominal aorta was isolated just between the branch of the renal and the iliac arteries, and an adjustable aortic clamp was placed around it to control renal perfusion pressure (RPP) during Ang II infusion and partial ischemia.

After the surgical procedure, the left jugular vein catheter was connected to the injection pump (New Era Pump System Inc., Farmingdale, NY, USA) for drug infusion. The carotid and femoral catheters were linked to a Powerlab System (ADInstruments, Australia) to measure systolic blood pressure (SBP), mean arterial pressure (MAP), and RPP respectively [31]. Besides, the RBF was measured by using a flowmeter (T402, Transonic System Inc., Ithaca, NY14850, USA), and renal vascular resistance (RVR) was calculated by the RPP/RBF ratio.

### 2.4. Experimental Protocol

The animals with SBP ≥150 mmHg were divided into three experimental groups of sham, IR, and IPC + IR. Following surgical procedures, the animals were allowed to stabilize and achieve equilibrium condition at least for 30 min. The SBP, MAP, RPP, RBF, and RVR were determined as baseline data which were obtained over the last 5 min of equilibrium that was called the “control” phase.

After the control phase, the experiment was continued according to the type of experimental group. In the sham group; the animals were subjected to the surgery process without IR or IPC. In the IR group, the RPP was reached to about 25 mmHg by using an adjustable occluder and was considered as partial kidney ischemia. Therefore, IR induced by controlling RPP in the range of 25 ± 3 mmHg by tightening the abdominal aortic clamp for 45 min. In the IPC group, IPC induced by two 5 min cycles of partial ischemia and 10 min reperfusion was performed before partial kidney ischemia for 45 min. The mean values for MAP, RPP, RBF, and RVR were measured during partial kidney ischemia called the “Ischemia” phase.

After 45 min of partial kidney ischemia, reperfusion was allowed by loosening the clamp, and the mean values for MAP, RPP, RBF, and RVR were measured at 1–3 min after beginning of reperfusion called the “Reperfusion1” phase.

Then, the antagonist (A779) or its vehicle (saline) was started to infuse with a microsyringe infusion pump (New Era Pump System Inc. Farmingdale, NY, USA) at 1–3 min after the beginning of reperfusion. Thirty minutes later, the data for MAP, RPP, RBF, and RVR were determined and were considered as an antagonist/vehicle effect called the “treat” phase. The A779 (Bachem Bioscience Inc., King of Prussia, PA, USA) was injected with a bolus dose of 50 *μ*g·kg^−1^ followed by continuous infusions of 50 *μ*g·kg^−1^ [25] using a microsyringe infusion pump.

### 2.5. Ang II Infusion

At 30 min after A779 or its vehicle administration, Ang II was infused in different doses of 30, 100, 300, and 1000 ng.kg^−1^·min^−1^, while the vehicle or A779 infusion continued until the end of the experiment. Each dose of Ang II was injected for 15 min. The data were recorded during the last 3–5 min of each dose of Ang II administration that was considered as a vascular response to Ang II infusion.

Finally, the rats were humanely sacrificed by anesthetic overdose of urethane (about 5 times of normal anesthetic dose; Merck, Germany) infused via a left jugular vein catheter, and the left kidneys were removed and weighed immediately.

### 2.6. Statistical Analysis

The data are presented as mean ± SEM, and the analysis was performed using SPSS version 22 software. Comparison of the MAP, RPP, RBF, and RVR between sham, IR, and IPC + IR groups in control, ischemia, reperfusion1, and treat phases was performed using one-way ANOVA followed by the LSD post hoc test. Responses to graded Ang II administration were analyzed by ANOVA for repeated measures followed by the LSD post hoc test. The *P* value ≤ 0.05 for the effect of graded Ang II (*P*_dose_), the comparisons between groups (*P*_group_), and the interaction between treatment and groups (*P*_dose×group_) was considered as significant.

## 3. Results

### 3.1. The Hemodynamic Parameters before Ang II Infusion

**Figure 1:**
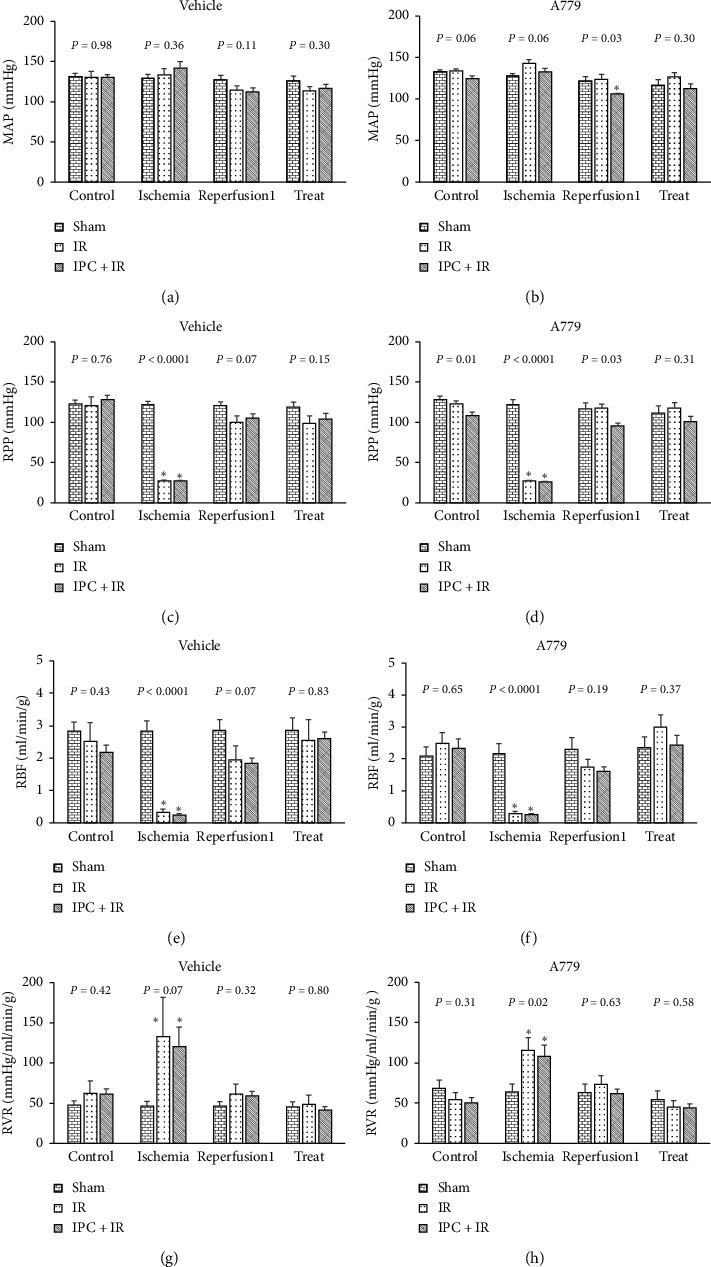
The hemodynamic parameters before Ang II administration in sham, IR, and IPC + IR groups in control, ischemia, 1–3 min postreperfusion (reperfusion1), and 30 min after antagonist/vehicle infusion (treat) phases. Data are presented as mean ± SEM. The *P* values were derived from one-way ANOVA. MAP: mean arterial pressure, RPP: renal perfusion pressure, RBF: renal blood flow, RVR: renal vascular resistance. ^*∗*^Significant difference from the sham group (*P* < 0.05).

There were 4 phases of measurements before Ang II administration: control, ischemia, reperfusion1, and treat (Figure 1). MAP, RPP, and RBF, and RVR normalized to left kidney weight were compared between sham, IR, and IPC + IR groups which received A779 or its vehicle.

Although there were observed statistical differences in RPP, RBF, and RVR between sham, IR, and IPC + IR during ischemia, no significant differences were detected between the groups in control and treat phases (Figure 1). These data revealed that the antagonist or vehicle had no significant effect on hemodynamic parameters before Ang II administration.

### 3.2. The Hemodynamic Response after Ang II Infusion

**Figure 2:**
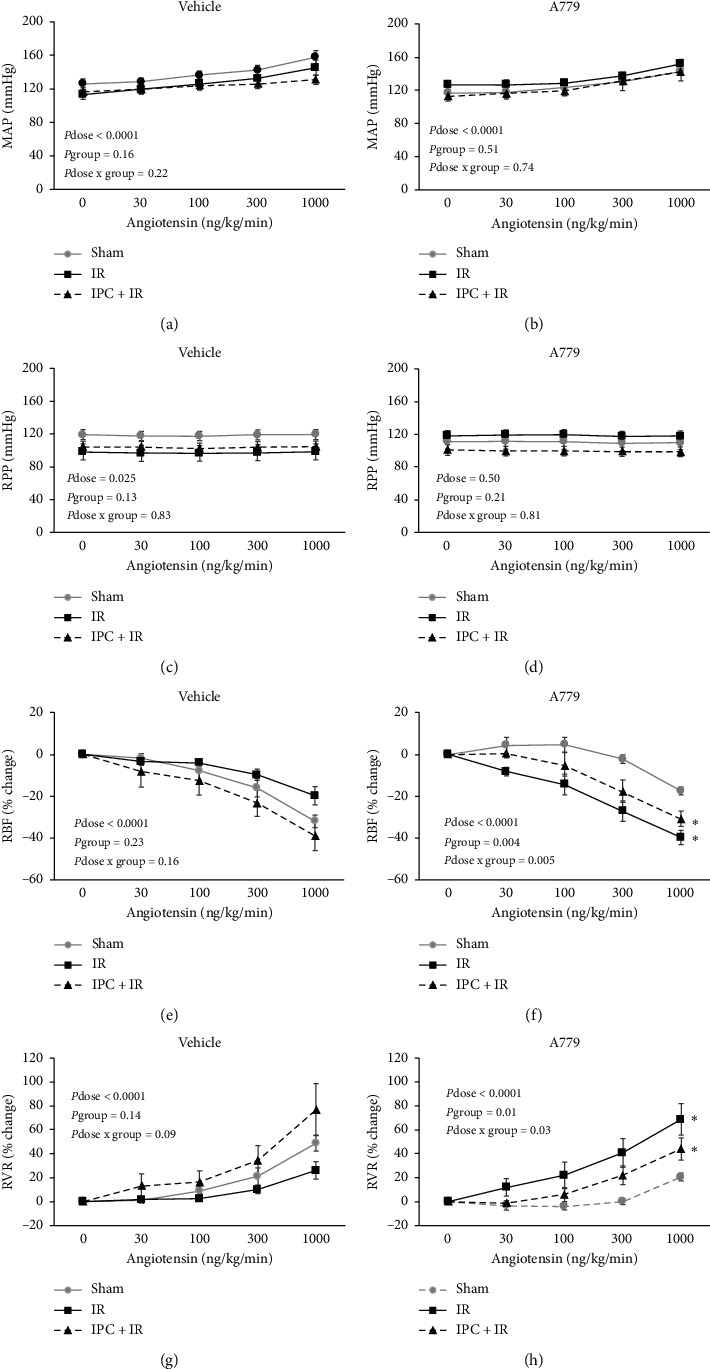
The hemodynamic parameter responses to Ang II infusion. Mean arterial pressure (MAP), renal perfusion pressure (RPP), renal blood flow percentage change (RBF%), and renal vascular resistance percentage change (RVR%) to graded Ang II infusion in sham, IR, and IPC + IR groups treated with A779 or its vehicle. Data are shown as mean ± SEM. The *P* values were derived from repeated measure ANOVA. ^*∗*^Significant difference from the sham group (*P* group < 0.05).

The intravenous infusion of graded Ang II resulted an increase of MAP in a dose- related manner in sham, IR, and IPC + IR groups receiving either A779 or its vehicle (Figure 2). However, no significant differences were detected between the groups.

As mentioned before, RPP was kept constant by manipulation of the aortic clamp during Ang II injection. Therefore, no alteration in RPP by Ang II administration was expected. However, a little increase in RPP was detected in the sham group which received the vehicle which was not considered to be important.

The percentage change of RBF (RBF%) response to graded Ang II infusion in sham, IR, and IPC + IR groups which received the vehicle for antagonist decreased dose dependently (*P*_dose_ < 0.0001), but no significant difference in RBF% response to graded AngII infusion was detected between the groups. However, when MasR was blocked with A779, a significant difference between sham and other groups in the RBF% response to Ang II administration was observed (Figure 2). For example, infusion of Ang II at dose 1000 ng·kg^−1^·min^−1^ resulted in RBF% to decrease from the baseline to 17.5 ± 1.9%, 39.7 ± 3.8%, and 31.0 ± 3.4% in sham, IR, and IPC + IR groups which received A779, respectively.

Infusion of graded Ang II increased the percentage change of RVR (RVR%) in sham, IR, and IPC ± IR groups treated with the vehicle in a dose-dependent manner (*P*_dose_ < 0.0001), but no significant difference in RVR% response to graded AngII infusion was detected between the groups. However, when MasR was blocked with A779, a significant difference between sham and other groups in the RVR% response to Ang II administration was observed (Figure 2). For example, infusion of Ang II at dose 1000 ng·kg^−1^·min^−1^ resulted in RVR% to increase from the baseline to 20.6 ± 3.1%, 68.8 ± 13.2%, and 44.1 ± 9.2% in sham, IR, and IPC + IR groups which received A779, respectively.

## 4. Discussion

The main findings of this study indicated that MasR blockade increased RBF and RVR responses to graded Ang II infusion after kidney partial IR with and without IPC in 2K1C hypertensive rats. It was also found that IPC did not provide a significant effect in RBF and RVR response to AngII whether MasR was blocked or not.

There are three items that influenced the results of this study including RAS, hypertension, and IR. RAS and its components play an important role in the pathogenesis of AKI induced by IR injury [17, 18, 32]. After 45 min of ischemia followed by 4 h of reperfusion, the increase of renal Ang II and the decrease of renal Ang 1–7 were detected [12, 22]. In rats with IR injury, AT_2_R expression has been observed in different parts of the renal tubule and glomerulus [22], and the renal expression of MasR increased [33]. Furthermore, renovascular hypertension affects the expression of RAS receptors and function. For example, MasR decreased in clipped kidneys of 2K1C rats [19]. The distribution of MasR in different parts of the kidney supports the contribution of the MasR in the regulation of renal function [15, 34]. So, MasR deficiency induces alterations in the hemodynamic parameters and function of the kidney. In MasR knockout animals, RBF decreased and RVR and glomerular filtration rate increased [35], and activation of the MasR declined AT_1_R-mediated responses [36]. It is reported that, in 2K1C rats, A779 limits the cellular signaling AT1R-mediated Ang II [37], and injection of 1 ng Ang II in the nonclipped kidney of the 2K1C induced a smaller renal vascular response [30]. Blockade of Ang 1–7 receptors worsened the course of hypertension and caused a significant reduction of renal hemodynamics in the nonclipped kidney of 2K1C hypertensive rats [38]. In our study, by MasR blockade, the RBF and RVR responses to Ang II were increased when compared with the sham group, and it was not consistent with others [30, 37, 38]. The difference possibly is related to IR and its effect on the kidney vascular system [39,40].

The beneficial effects of IPC are seen in kidneys [41–43]. IPC contributes to restoration of RBF following renal IR injury [44].On the contrary, no protective effect of IPC against IR was reported by others [45]. In addition, repeated renal IPC did not show an additional protective effect against renal IR when compared with a single cycle of IPC [46]. In the current study, IPC did not indicate a significant effect in RBF and RVR responses to AngII, whether MasR was blocked or not, which is consistent with others [45]. Another possibility is that the effect of partial IPC may be limited due to vascular alteration in the hypertension condition [47]. There was a limitation in this study which could also change the outcome. In IPC, the renal artery was not completely occluded, and it included two cycles of partial ischemia. Therefore, the two cycles of short partial ischemia may not be enough to protect the kidney against long time of partial ischemia single cycle of IPC [46]. Finally, the finding of this study may be implemented whenever the kidney is subjected to hypoperfusion [48]. For example, cardiac surgery induces kidney hypoperfusion and increases the risk of AKI [49, 50] while hypertension itself is a risk factor for AKI [51]. There are many challenges for therapeutic strategies related to RAS receptor (AT_1_R) blocker and enzyme (ACE) inhibitor to reduce kidney complications following cardiac surgery [49], and these strategies may increase the important role of the Ang1–7-MasR axis. Therefore, it seems that regulation of MasR activity can be implemented clinically to reduce ischemic complications during hypoperfusion.

## 5. Conclusions

Kidney IR promoted the RBF and RVR responses to Ang II administration in 2K1C hypertensive rats when MasR was blocked. This effect may be related to vascular function alteration in the kidney after partial IR.

## Data Availability

Data will be provided on request by the corresponding author.
